# Protocol for sub-mucosal orthotopic injection of organoids into murine colon to study tumor growth and metastasis

**DOI:** 10.1016/j.xpro.2025.103887

**Published:** 2025-06-12

**Authors:** Sura Atatri, Marianne Meyers, Eliane Klein, Valentina Brunner, Markus Tschurtschenthaler, Elisabeth Letellier

**Affiliations:** 1 Department of Life Sciences and Medicine, University of Luxembourg, L-4367 Belvaux, Luxembourg; 2 Center for Translational Cancer Research (TranslaTUM), School of Medicine and Health, Technical University of Munich, 81675 Munich, Germany; 3 Institute of Experimental Cancer Therapy, Klinikum Rechts der Isar, School of Medicine and Health, Technical University of Munich, 81675 Munich, Germany; 4 Division of Translational Cancer Research, German Cancer Research Center (DKFZ) and German Cancer Consortium (DKTK), 69120 Heidelberg, Germany

**Keywords:** Cancer, Health Sciences, Model Organisms, Organoids

## Abstract

Orthotopic mouse models of colorectal cancer (CRC) better recapitulate the physiological processes of tumor development and metastatic dissemination. Here, we provide a protocol for colonoscopy-guided transplantation of organoids into the murine colon. We describe the steps for preparing mouse organoids, equipment, and mice for injections, as well as performing colonoscopy-guided mucosal injections and providing subsequent care. This model can be used to investigate various experimental setups, including survival, metastatic potential, and the effects of treatments.

For complete details on the use and execution of this protocol, please refer to Felchle et al.^1^

## Highlights

• Steps for orthotopic injections of organoids into the mouse colon
• Guidance on organoid lines and mouse strains
• Instruction for successful injections
• Advice on tumor growth kinetics

## Abstract (editor highlights)

Publisher’s note: Undertaking any experimental protocol requires adherence to local institutional guidelines for laboratory safety and ethics.

## Graphical abstract

**Figure:**
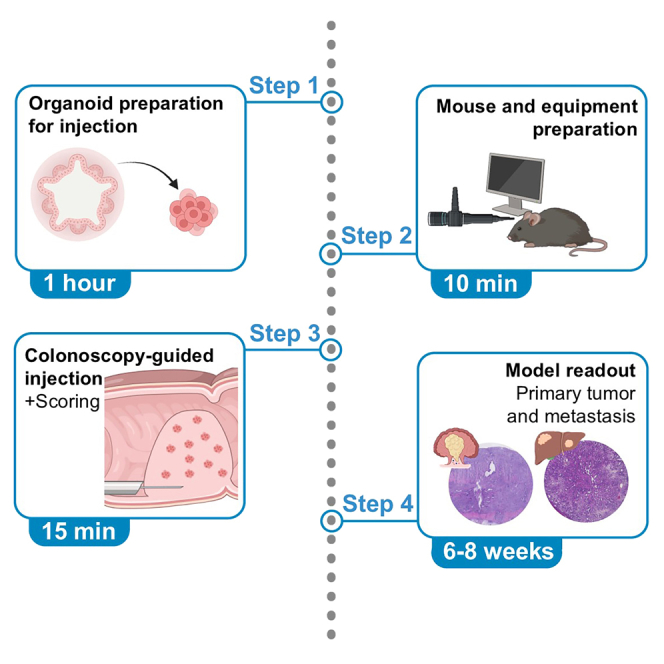

## Before you begin

The protocol below describes the steps for using both small and large mouse intestinal organoids and engrafting them into syngeneic immunocompetent C57BL/6J mice. However, it could be applied to a variety of organoid lines as well as mouse strains, provided that the organoid lines and mouse strains are compatible.

**CRITICAL:** Ensure that all animal work is approved by the necessary internal animal welfare committees of your research institution and dedicated authorities.

***Note:*** This protocol does not provide a detailed overview of organoid cultures and assumes already established cultures in your research laboratory. For complete details on organoids generation and cultivation, please refer to Felchle et al.^1^

***Note:*** This protocol requires two people (an *Operator* and an *Assistant*) specifically for the orthotopic injection part of the protocol. Additionally, we strongly recommend adequate training to reduce injection variability.

**CRITICAL:** It is important to thoroughly sterilize and prepare all the necessary equipment ahead of time.

The section below outlines steps for preparing the workspace.

1. Thaw Corning Matrigel Basement Membrane Matrix (hereafter simply referred to as Matrigel) on ice, preferably at 4°C for 2–3 h prior to starting.
2. Ensure sufficient isoflurane is available for the duration of the procedure by refilling to the maximum level prior to starting.
3. Apply eye ointment to prevent corneal drying caused by general anesthesia and airflow through the nose cone. Additionally, the opaque eye ointment blocks bright light, which could be a hindering stimulus, thereby allowing for better anesthesia quality.
4. Turn on the heating pads and set them to 37°C to minimize the risk of hypothermia. Place a piece of tissue between the mouse and the pad for added comfort and safety. Heating pads should be used throughout the duration of the procedure to prevent hypothermia.

### Institutional permissions

All animal experiments were performed in compliance with European guidelines for the care and use of laboratory animals, including EU Directive 2010/63/EU and the Grand-Ducal Regulation of 11 January 2013 on the protection of animals used for scientific purposes. The studies were approved by the relevant ethics committees and legal entities, including the Animal Experimentation Ethics Committee at the University of Luxembourg (AEEC) and the Ministry of Agriculture, Food and Viticulture (LUPA 2020/31), as well as the Institutional Animal Care and Use Committees (IACUC) of the Technische Universität München and the Regierung von Oberbayern (animal protocol number: ROB-55.2-2532.Vet_02-19-186).

### Mouse housing

**Timing: Variable**

The below section specifies the recommended housing conditions for the mice during the experiment.

5. House mice in an approved specific pathogen-free (SPF) facility under controlled conditions.

***Note:*** Relative humidity of 40–70%, temperature at 22°C, and a 12 h dark/light cycle.

6. Provide mice with food and water ad libitum, as well as sufficient bedding and enrichment, such as tunnels and gnawing sticks.

### Equipment preparation

**Timing: 10 min**

**Figure 1:**
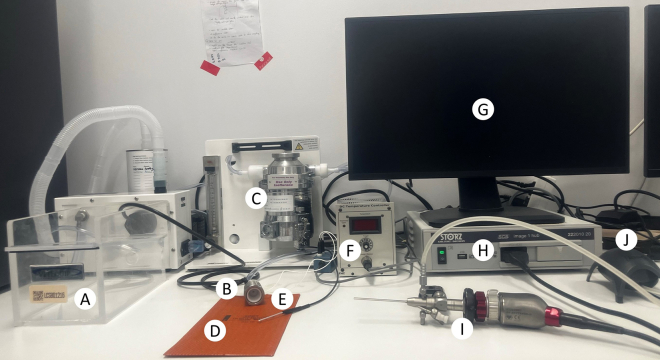
Endoscopy setup (A) Isoflurane induction chamber (B) Isoflurane nose cone (C) Isoflurane source (D) Heating pad (E) Temperature probe (F) Thermometer (G) Monitor (H) Colonoscopy console (I) Camera head and endoscope (J) Air pump.

**Figure 2:**
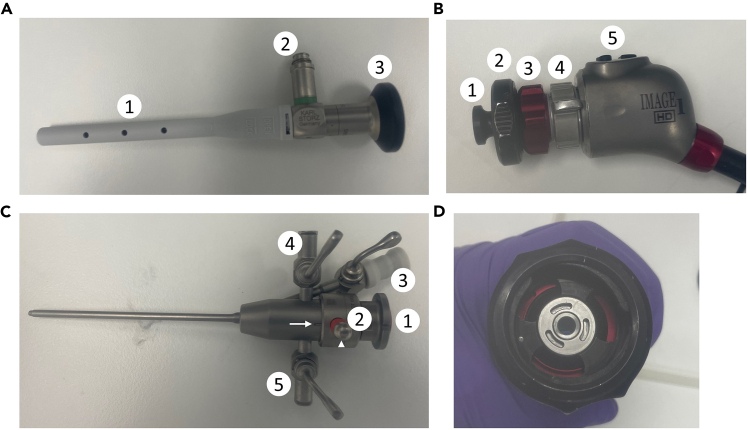
Endoscope setup and components (A) Endoscope, 1: plastic cover, 2: light port, 3: camera port. (B) Camera head, 1: lens cap, 2: black dial, 3: focus dial, 4: magnification dial, 5: camera buttons. (C) Examination sheath, 1: obturator, 2: security level, 3: injection needle inlet, 4: air valve, 5: extra air valve. (D) Camera lens on the camera head with the lens cap removed, connection site of the telescope camera port.

**Figure 3:**
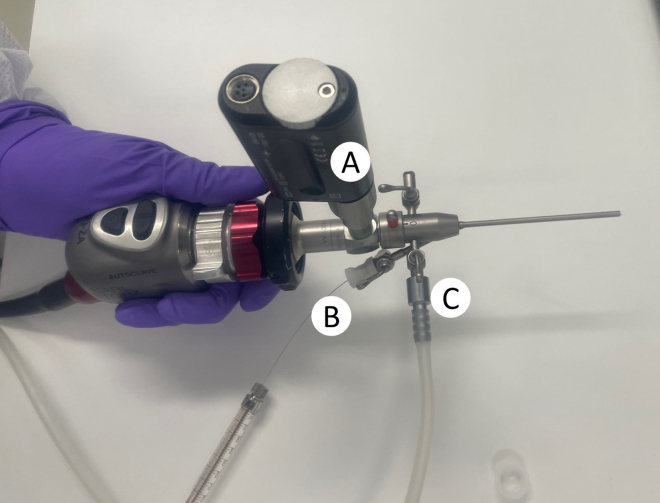
The final, completely assembled camera and endoscope for colonoscopy and/or injection (A) Light source (B) Hamilton injection needle (C) Air valve and air pump connector.

The section below details the steps required for setting up the adapted Image 1 Hub system from Karl Storz (Figure 1). The components of the endoscope are described in Figure 2, while the final assembled setup of the camera and endoscope are depicted in Figure 3. The setup should be assembled and checked for operationality prior to proceeding with the protocol.

7. Carefully remove the plastic cover (Figure 2A.1) of the telescope by gently twisting it and then pulling it straight out.
8. Remove the camera head lens cap (Figure 2B.1) from the camera head by turning the black dial (Figure 2B.2) 90° clockwise, then pulling the cap off while holding the black dial at a 90° angle.

***Optional:*** For optimal image quality you can clean both the telescope camera port (Figure 2A.3) and the camera lens on the camera head (Figure 2D) with a Kimtech Science Delicate Task Wipers.

9. Again, turn the black dial on the camera head (Figure 2B.2) 90° clockwise to carefully align the telescope such that the light port (Figure 2A.2) is positioned in line with the camera buttons (Figure 2B.5) on the top of the camera head. To lock the telescope in place, release the black dial carefully and ensure the telescope is securely held in position.
10. Remove the obturator from the examination sheath (Figure 2C.1) by releasing the security lever (Figure 2C.2) to 90° and gently pulling the obturator straight out.
11. Attach the obturator to the telescope and refasten the security lever, ensuring that the lever and the indentation on the examination sheath are aligned (as indicated by the arrows in Figure 2C).
12. Gently screw on the light source to the telescope light port (Figure 2A.2).
13. Gently screw on the air pump to the air valve on the examination sheath (Figure 2C.4) and ensure that the extra air valve (Figure 2C.5) is closed (i.e. the lever is perpendicular to the inlet tube).
14. The camera and endoscope should now be fully set up and resemble Figure 3 (without the injection needle).
15. Turn on the endoscopy system (Image 1 HUB) (Figure 1), the light, and the air pump.
16. Test if there is airflow coming through the endoscope, by placing the tip of it inside a 15 mL falcon tube filled with PBS (1X) at room temperature. Set the airflow to have 1 bubble per second by adjusting the lever on the air valve.
17. Navigate the camera settings to select the white balance option, ensuring the endoscope is pointed at a white surface. Proceed only after the white balance has been successfully completed.
18. Place a USB device in the endoscopy system (Image 1 HUB) to save captured images and/or recorded videos.

## Key resources table

**Table:** 

REAGENT or RESOURCE	SOURCE	IDENTIFIER
**Chemicals, peptides, and recombinant proteins**		

PBS (1X) pH 7.4	Gibco	10010-023
Matrigel basement membrane matrix, growth factor reduced (GFR), phenol red-free, LDEV-free	Corning	356231
Cell recovery solution	Corning	354253
Advanced DMEM/F-12	Gibco	12634010
GlutaMAX supplement (100X)	Gibco	35050061
N-2 supplement (100X)	Gibco	17502048
B-27 supplement (50X), serum free	Gibco	17504044
Penicillin-streptomycin (10,000 U/mL)	Gibco	15140148
ROCK inhibitor (Y-27632)	STEMCELL Technologies	72304
TrypLE express enzyme (1X), no phenol red	Gibco	12604013
Isoflurane CP	CP-Pharma	1214
Bepanthen eye and nose ointment	Bepanthen	01578681

**Experimental models: Cell lines**		

MT577 T1-2	Provided by Markus Tschurtschenthaler^1^	Derived from a *Kras*^LSL-G12D^ mouse intestinal adenocarcinoma G2 with the following genomic characteristics: *Trp53*^WT^, *Cdkn2a* (p16^INK4A^/p19^ARF^) deletion, *Ctnnb1* missense mutation (p.Ser37Phe), and chromosome 6 amplification including *Kras*^G12D^.
APKS mScarlet (*Apc*^*KO*^, *Trp53*^*KO*^, *Kras*^*G12D*^, *Smad4*^*KO*^ mutated)	Provided by Semir Beyaz^2^	In short, organoids were isolated from the healthy colonic epithelium of C57Bl/6 *Kras*^LSL-G12D^; *Trp53*^flox/flox^ mice following adenoviral Cre-mediated recombination. They were then treated with a lentivirus expressing miR-30 short hairpin (sh)RNA against *Apc* and SIINFEKL fused to the fluorophore mScarlet (mScarlet^SIIN^). Furthermore, deletion of *Smad4* was achieved by CRISPR-Cas9 editing.
L-WRN cell line	ATCC	CRL-3276

**Experimental models: Organisms/strains**		

C57BL/6J mice	8- to 12-week-old male mice, from an in-house maintenance breeding regularly refreshed with mice purchased from The Jackson Laboratory.	

**Other**		

DC temperature controller	FHC	40-90-8D
WATLOW heating pad	WATLOW	050100C1
Oxygen concentrator	Zyklusmed	K5BW
Anesthetic vaporizer	Harvard Apparatus	34-1040
Activated charcoal filter	VaporGuard	931401
Endoscopy system – Image 1 HUB	Karl Storz	222020 20
Endoscopy system – camera head	Karl Storz	H3-ZA
Light	Karl Storz	11301 DF
Endoscope – HOPKINS Telescope (0˚, 1.9 mm rigid endoscope)	Karl Storz	27301AA
Endoscope – HOPKINS examination sheath, 9 Fr.	Karl Storz	27033DK
Air pump	JBL	ProSilent a100
Hamilton syringe (100 μL)	Hamilton	80630
Hamilton transfer needle	Hamilton	7770-02
Hamilton injection needle (33-gauge, small hub-RN needle, 16-inches long, point style 4, 45-degree bevel)	Hamilton	7803-05
Gavage needle	Fine Science Tools (F.S.T)	18060-20
Kimtech Science delicate task wipers	Kimberly-Clark	7558

## Materials and equipment

### Culture medium for organoid cell lines

**Table:** APKS

Reagent	Final concentration	Stock concentration	Volume (mL) to add to 500 mL
Advanced DMEM/F-12	–	–	480 mL
GlutaMAX supplement	1X	100X	5 mL
N-2 supplement	1X	100X	5 mL
B-27 supplement	1X	100X	5 mL
Penicillin-Streptomycin	100 U/mL	10,000 U/mL	5 mL

Store at 4°C for up to 1 month.

**Table:** MT577 T1-2

Reagent	Final concentration	Stock concentration	Volume to add to 50 mL
Advanced DMEM/F-12	–	–	25 mL
L-WRN conditioned medium^3^	50%	100%	25 mL
ROCK inhibitor Y-27632 (*added for thawing, 2-3 days after passage, and injection*)	10 μM	10 mM	5 μL

Store at 4°C and use it within 1–2 weeks.

## Step-by-step method details

### Preparation of mouse organoids for injection

**Timing: 1 h**

The section below outlines the steps to be carried out in a cell culture setting for the preparation of the organoids for injection.

**CRITICAL:** Ensure the organoids have been tested for mycoplasma prior to transplantation.

**Figure 4:**
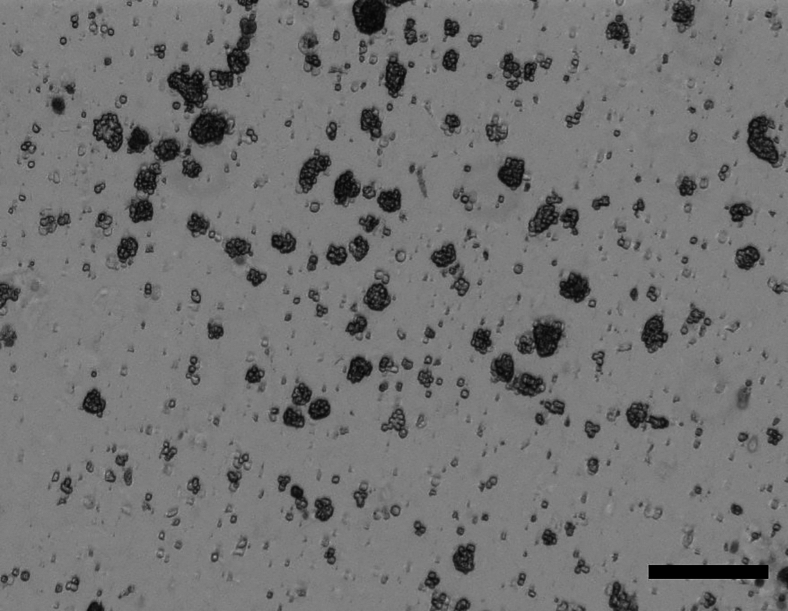
Organoids fragments after mechanical dissociation Scale bar is 300 μm.

***Note:*** Chemical dissociation (i.e., TrypLE Express Enzyme) might be required depending on the organoid line.

1. Wash organoid domes once with 500 μL of PBS (1X) at room temperature.
2. Overlay domes with 350 μL of Cell Recovery Solution (Corning) and incubate on ice for 30 min.
3. Mechanically dissociate organoids into fragments (∼10–20 cells per fragment) with a pre-wet P20 stacked on a P1000 tip. The dissociated organoids should resemble those shown in Figure 4.

4. Collect organoid fragments suspension in a falcon tube and centrifuge at 350 g for 5 min. Resuspend in the corresponding volume of injection solution.
  a. Inject ∼150 organoid fragments (∼10–20 cells per fragment) per injection, up to three injections per mouse depending on organoid line, using a volume of 100 μL per injection.
    ***Note:*** As reference for the APKS and the MT577 T1-2 organoids, this was roughly three confluent 50 μL Matrigel domes (at days 3 and 4 of culture, respectively) required per mouse (accounting for excess).
    ***Note:*** Depending on the mutational profile, and consequently, the growth potential and aggressiveness of the model, the number of injections and the fragments count may need to be adjusted.
  b. Injection solution: PBS (1X) + 10% Matrigel + 10 μM Rock-Inhibitor (Y-27632).
5. Store organoid fragments in injection solution on ice until orthotopic injection.

**CRITICAL:** Minimize the time the organoids remain in the injection solution and on ice to ensure optimal organoid viability; we recommend limiting this to no more than 3 h.

### Preparing mice for injection

**Timing: 10 min per mouse**

The section below outlines the steps required to prepare the mouse for the subsequent injections, which are to be conducted in an appropriate animal facility.

6. Turn on the anesthetic vaporizer and the corresponding filter and oxygen concentrator.
7. Make sure the Isoflurane induction chamber is completely closed and set the level of isoflurane to 5%. Wait a few minutes for the induction chamber to fill up with the isoflurane.
8. Anesthetize one mouse at a time by placing it inside the isoflurane chamber. Wait until the mouse is fully anesthetized (1–2 min), indicated by a slower breathing rate and absence of movement.
9. Remove the anesthetized mouse from the isoflurane induction chamber and place it on top of the heating pad in a prone position.

**CRITICAL:** Make sure the isoflurane nose cone covers the head of the mouse, and that sufficient eye ointment is applied.

10. Lower the level of isoflurane to a maintenance level of 2% and adjust it if needed during the procedure.

***Note:*** Alternative anesthesia methods, such as injectable anesthesia, can be used and must be approved by your institutional animal welfare committee. Options include ketamine (100–150 mg/kg) combined with medetomidine (0.75–1 mg/kg), administered subcutaneously (s.c.) or intraperitoneally (i.p.). Other injectable anesthetics may also be suitable. However, compared to inhalation anesthesia, injectable anesthesia typically results in longer durations of anesthesia and requires careful consideration of post-procedure heating support.

11. Capture an image of the mouse identifier so it becomes easier to track the captured colonoscopy images later.
12. Perform an anal enema using a straight feeding needle connected to a syringe filled with ∼1 mL of PBS (1X) at room temperature and flush the colon to remove stool.

### Colonoscopy-guided mucosal injection

**Timing: ∼15 min per mouse**

The section below outlines the steps for the injections of the above prepared organoids into the mice.

13. Prepare the mice as outlined above for colonoscopy following step 6 to 12.

***Note:*** From this point onwards, it is recommended to work with two people. An “*Operator*” that is managing the endoscope and performing colonoscopy, and an “*Assistant*” that is responsible for the orthotopic injection. We recommend ample training before, of both the “*Operator*” and the “*Assistant*”, to allow for consistent injections and reduce variability within experimental cohort.

**Figure 5:**
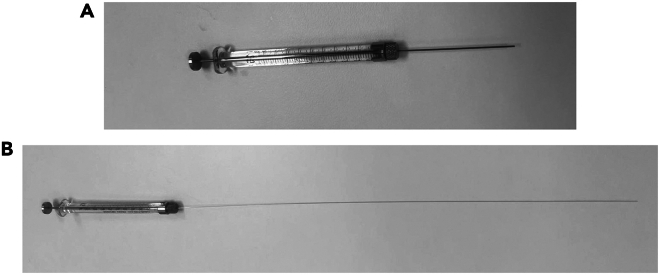
Transfer and injection needles Hamilton syringe with either the (A) Transfer needle or (B) Injection needle.

***Note:*** Ensure that the organoid fragments suspension is well resuspended before drawing up for injection for equal distribution of organoid fragments per injection.

14. *Assistant:* Attach the transfer needle to the Hamilton syringe (Figure 5A) and carefully draw up 100 μL of the organoid fragments suspension.

15. *Assistant:* Carefully unscrew the transfer needle and attach the injection needle to the syringe (Figure 5B).

***Note:*** Ensure that there is no leakage in your syringe setup.

**CRITICAL:** After replacing the transfer needle with the injection needle, gently push the organoid injection suspension to the tip of the long injection needle to prevent introducing air during the injection.

16. *Operator:* Insert the endoscope approximately 3 cm into the distal colon, up until you reach the “natural curve”.

***Note:*** This positioning provides sufficient space for additional injections, if needed. The injection should be made on the ventral side of the colon wall. For rectal injections, insert the endoscope tube only about 1 cm.

17. *Assistant:* Insert the injection needle into the inlet of the examination sheath (Figure 2C.3 or Figure 3B).

**CRITICAL:** Make sure that the inlet lever is open (aligned parallel to that of the tube) to allow a clear passage for the needle. Any obstruction could blunt the needle and compromise the success of the injection.

**Figure 6:**
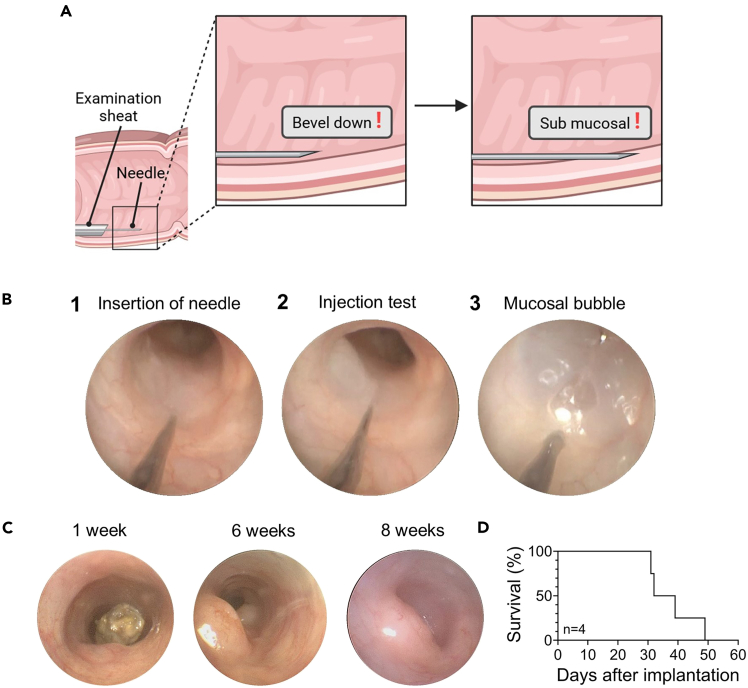
Overview of sub-mucosal orthotopic injection into the murine colon (A) Schematic representation of the needle orientation and depth of injection in the murine colon. Created in BioRender. Meyers, M. (2025) https://BioRender.com/e38i854. (B) Subsequent steps of orthotopic injection in murine colon. 1: Insertion of needle, 2: Injection test, 3: Mucosal bubble. (C) An example of tumor growth as follows via colonoscopy over the duration of an experiment (Two injections of MT577 T1-2, ∼150 organoid fragments per injection in C57BL/6J mice). (D) Survival of mice in days after orthotopic injection of MT577 T1-2 organoids (n = 4 mice).

18. *Assistant:* Inject organoid fragments into the submucosa of the murine colon (Figure 6).

**CRITICAL:** The bevel of the needle should face downward toward the wall of the colon.

***Note:*** Prior to administering the full injection volume, a preliminary test injection of approximately 10 μL should be performed to ensure the absence of leakage into the intestinal lumen. Upon confirmation of the correct placement, the full injection volume (100 μL) should be delivered in a single, continuous motion, ensuring completion within less than one second. Proper submucosal injection will induce a visible elevation of the submucosa, forming a localized “bubble” without diffusion into the lumen (Figure 6B and Methods Video S1). If a leakage is observed, abort injection and relocate injection site.

***Optional:****Operator:* If it is planned to perform a second injection, move the endoscope < 1 cm distally. *Assistant:* Repeat steps 14 to 18.

**CRITICAL:** The *Assistant* should always retract the injection needle before the *Operator* repositions the endoscope to prevent any injury to the animal.

Methods Video S1. Example of a successful sub-mucosal orthotopic injection into the murine colon, related to step 18

### Aftercare and maintenance

**Timing: Throughout the duration of the experiment**

The section below outlines the steps required for following up on the mice throughout the duration of the experiment.

19. After the injection, remove the endoscope from the mouse.
  a. Take the mouse off the nose cone and return it to its cage.
  b. Place a heating pad set at 37°C under half of the cage to allow for full recovery of the animal.

***Note:*** Ideally, place the animal inside a tunnel or cover it with some tissue paper to reduce light exposure and minimize stress during the wake-up process.

20. After the injection, grade the injection efficiency using the rubric provided in Table 1.

**Table 1:** Scoring rubric of sub-mucosal orthotopic injections into the murine colon based on two criteria: Bubble size in relation to the lumen (lumen filled in percentage [%]), and whether the bubble remained post injection or was reabsorbed within a few seconds

	Bubble remained	Bubble reabsorbed
Bubble filled 75–100% of the lumen	A	a
Bubble filled 50–75% of the lumen	B	b
Bubble filled <50% of the lumen	C	c

***Optional:*** If the duration of the injection procedure exceeded ∼3 h, consider plating the excess organoid fragments suspension to assess cell viability and culture behavior.

21. Mice should be monitored via colonoscopy throughout the experiment, following the same steps outlined in the “Preparing mice for injection” section.

**Figure 7:**
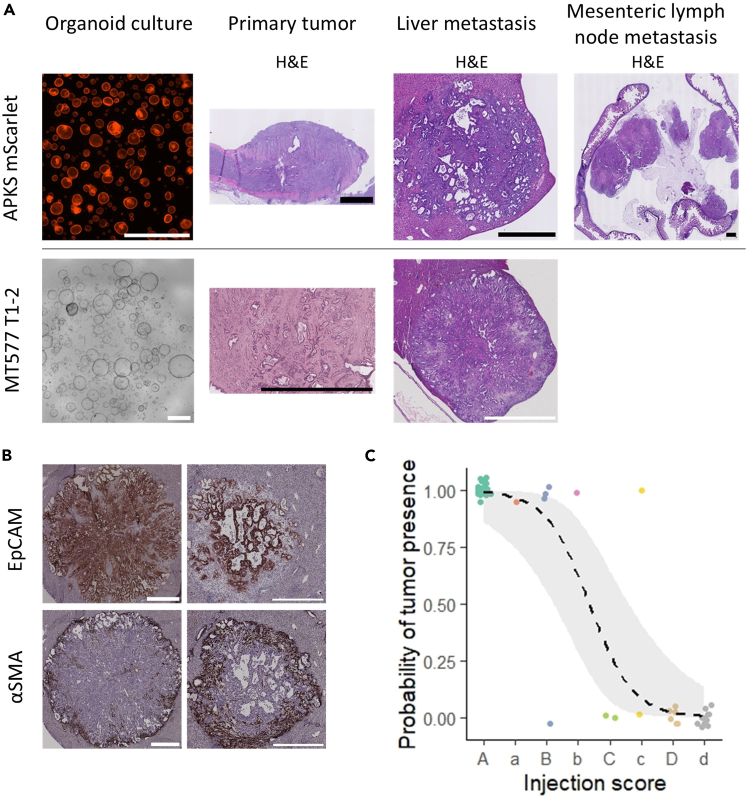
Examples of expected outcomes of sub-mucosal orthotopic injection of two different organoid lines, MT577 T1-2 and APKS mScarlet, into C57BL/6J mice (A) Representative bright-field and fluorescence images of organoids, and H&E staining of a colon tumor after orthotopic transplantation and subsequent distant organ metastases. Scale bars are 1000 μm. (B) Representative images of αSMA and EpCAM staining of activated fibroblasts and epithelial cells, respectively, in liver metastasis of MT577 T1-2-injected C57BL/6J mice. Scale bar represents 500 μm. (C) An example of an experimental cohort showing probability of tumor presence versus injection score. Dashed line and gray area represent GLM fitted curve and confidence interval, respectively. Analysis of deviance of the binomial generalized linear model (GLM) testing the significance of the injection score on the probability of generating a tumor (n = 49 injections, Pr > ChiSq, 3.718e-12, ∗∗∗).

***Note:*** The frequency of monitoring should be determined in agreement with your animal welfare and/or ethics committee and should reflect the severity of the model, which may vary depending on the organoid and mouse lines used. Examples of expected outcome are illustrated in Figures 6C and 6D and Figure 7A.

## Expected outcomes

### Primary tumor and metastasis formation

The duration and outcomes of the model—including tumor growth, tumor count, and sites of metastases—are highly dependent on the organoid line, its respective mutational profile, and the mouse line used. Tumor growth can be assessed via colonoscopy and scored based on the percentage of luminal obstruction, as significant obstruction may lead to the development of an ileus (Figure 6C). In general, it is accepted that the humane endpoint (HEP) for mice is reached when the primary tumor obstructs more than 75% of the colon lumen. The definition of the humane endpoint should be reviewed and approved by the animal welfare committee of your research institution and the relevant regulatory authorities. In our experiments, both MT577 T1-2 and APKS mScarlet-injected mice reached HEP within 8-11 week, as determined by colonoscopy showing 75% luminal obstruction. By this stage, mice had developed primary tumors and liver metastases (Figures 7A and 7B). For the APKS mScarlet organoids, mesenteric lymph node metastases were also observed in some cases (Figure 7A). It is important to note that the duration of an experiment can vary significantly depending on the organoid line, mouse strain, facility conditions, and additional treatments. For instance, the MT577 T1-2 organoids have been previously reported to reach HEP at 5-8 weeks,^1^ while a different line of APKS organoids has been found to form primary tumors by 8 weeks.^4^

### Characterization of the TME in the model

Various downstream assays can be employed to characterize the tumor microenvironment (TME). Among the most used methods is flow cytometry, which enables the analysis of immune and stromal cell populations using different antibodies. These can be surface-based or intracellular, allowing not only for the identification of cell presence but also the assessment of their activation or inhibition states. Additionally, histological staining can be performed on tissue samples to visualize the stromal compartment and immune cell infiltration within the TME. As an example, we present the staining of α smooth muscle actin (αSMA), a marker for activated fibroblasts within metastatic tissue (Figure 7B).

### Application of the method using other mouse strains

The primary objective of this protocol was to demonstrate the orthotopic injection of organoids in immunocompetent mice to study metastasis in the context of a fully functional immune system. Therefore, we used the C57BL/6 strain, as it matches the genetic background of the organoids available in our biobank. It is crucial that researchers ensure compatibility between the organoid and the host strain before initiating an experiment to prevent graft rejection. Additionally, we have successfully achieved injection and engraftment of organoids in immunocompromised NOD.Cg-*Prkdc*^scid^ *Il2rg*^tm1Wjl^/SzJ (NSG) mice. This immunocompromised model is particularly suitable for studies involving human organoids when TME interactions are not the primary focus.

## Limitations

This technique demands significant effort to develop the expertise required for consistent and precise injections due to their intricate nature. Additionally, the equipment necessary for this protocol is highly specialized and expensive. From a biological perspective, the model relies on the introduction of a bulk of cells (organoid fragments) at once, which makes it less representative of the early tumor initiation phases and may lack some of the microenvironmental components crucial to initial tumor formation.

Whereas the proposed model is ideal to study metastasis dissemination in CRC, it can also be adapted to study the early stages of tumorigenesis. For instance, early adenoma lines can be injected to analyze the transition from adenoma to carcinoma. Additionally, this model allows for the injection of organoids that have been genetically engineered *in vitro* to overexpress a specific oncogene implicated in early tumor development. These organoids can be designed with an inducible promoter controlling oncogene expression, which is activated only after successful engraftment into the colonic wall. Furthermore, tamoxifen can be orthotopically injected into the colonic wall of a transgenic mouse, locally triggering oncogene activation and enabling the study of tumor initiation and progression from the earliest stages to carcinoma formation at the specific injection site.

## Troubleshooting

### Problem 1

Difficulty obtaining the correct injection depth and specifically reaching the submucosal layer.

### Potential solution

The difficulty of injecting only submucosally may be coming from the lack of resistance of the colon wall. Try to increase the airflow slightly thereby increasing the resistance of the tissue. Alternatively, try adjusting the angle of injection (please refer to Figure 6A).

### Problem 2

Difficulty inserting the needle submucosally.

### Potential solution

If the needle is just “slipping” past the tissue, replace the needle as the needle may be dull. We recommend replacing the needle after every ten mice. Please remember the NOTE in step 17: “ensure that the inlet lever is open (parallel to that of the tube) to ensure clear passage of the needle. Any obstruction may blunt the needle and hinder successful injection.” Alternatively, consider increasing the air flow to increase colon wall resistance.

### Problem 3

Mouse “ballooning” post procedure.

### Potential solution

Reduce the airflow throughout the procedure, only increasing it during the injection to avoid excessive airflow overall (Reminder from the material and equipment setup section: “Test the airflow coming through the endoscope by placing it inside a 15 mL falcon tube filled with PBS. Adjust the airflow to produce one bubble per second by tightening the nozzle on the air pump tube.”). Alternatively, consult your veterinarian to ensure the anesthesia is sufficient. Inadequate anesthesia can prevent full muscle relaxation, causing the mouse to “trap” air, which may then move into the small intestine. Lastly, reevaluate the extent of the anal enema to ensure the cecum has not been affected as it could contribute to airflow passing through to the small intestine.

### Problem 4

No appearance of bubble post injection.

### Potential solution

Adjust the depth of injection. It could be that the injection was not done in the submucosa, but rather in the muscle layer (muscularis propria). NOTE: This would nonetheless lead to tumor formation but on the peritoneal side of the intestine rather than the luminal side. Alternatively, it could be that the injection solution is either leaking into the lumen during injection, meaning the depth of injection is not deep enough, or being lost at some point in the injection setup (refer to step 15). To assess injection depth or leakiness consider using an ink as an indicator during training sessions.

### Problem 5

No growth of a primary tumor.

### Potential solution

Assess the viability of the organoids post injection by plating the non-injected excess of injection solution as outlined in optional step 21. It would be expected that the re-seeded organoids would grow to a minimum of 70–80% of the original confluency of the organoid culture prepared for injection. However, if distant metastases are observed without primary tumor formation, reconsult your injection score as the injection might have perforated into the peritoneum or blood stream.

### Problem 6

The rapid growth of the primary tumor prevents the development of metastases, making it unsuitable if metastasis formation is the goal of the mouse model.

### Potential solution

Lower the cell count and/or lower injection number per mouse.

### Problem 7

Inconsistent results in terms of tumor growth.

### Potential solution

If the experimental setup allows, stratify mice evenly between experimental groups based on their injection score, please refer to step 20 and Table 1. As a suggestion, it would be fair to include only “A”, “a”, “B” and “b” scored injections into a study cohort. An example of an experimental cohort showing primary tumor incidence versus injection scores is shown in Figure 7C. Consider investing in adequate training to ensure having consistent outcome. As an example, an ink can be used to assess injection efficiency during training.

You are encouraged to write down any comments or incidents during the injection. Eventually they can explain inconsistent results.

## Resource availability

### Lead contact

Further information and requests for resources and reagents should be directed to and will be fulfilled by the lead contact, Elisabeth Letellier (elisabeth.letellier@uni.lu).

### Technical contact

Questions about the technical specifics of performing the protocol should be directed to the technical contacts, Sura Atatri (sura.atatri@uni.lu) and Marianne Meyers (marianne.meyers@uni.lu).

### Materials availability

This study did not generate new unique reagents.

### Data and code availability

This study did not generate or analyze datasets nor code.

## Acknowledgments

This research was funded by the Luxembourg National Research Fund (FNR): PRIDE19/14254520 (S.A.) and MFP20/15251414/MelCol-PFP (E.L.). It was further supported by an FNR and Fondation Cancer CORE/C20/BM/14591557 (E.L.); an FNRS-Télévie grant 7.4565.21-40007364 (M.M.); the Fondation du Pélican de Mie and Pierre Hippert-Faber under the aegis of the Fondation de Luxembourg, ‘Pelican Grant’ (M.M.); the European Union’s Framework Programme for Research and Innovation Horizon 2020 (Marie Skłodowska-Curie grant agreement no. 753058 [M.T.]); and the Deutsche Forschungsgemeinschaft (SFB1371 Microbiota signatures) project ID 395357507, P11 (M.T.). For the purpose of open access, and the fulfillment of the obligations arising from the grant agreement, the author has applied a Creative Commons Attribution 4.0 International (CC BY 4.0) license to any author-accepted manuscript version.

We would like to thank M. Schmoetten for her extensive help during the animal experiment, D. Cheung for his support in organoid cultures, and M. Gabola for the image acquisition support. We would like to thank S. Beyaz for providing the APKS mScarlet organoids. We are also grateful to D. Coowar for managing the rodent facility, as well as the veterinarian service (J. Behm) of the University of Luxembourg for their assistance and guidance in animal welfare during animal experimentation, and particularly for proofreading this protocol. This project was also supported by the Doctoral School in Science and Engineering (S.A. and M.M.) and the Department of Life Sciences and Medicine at the University of Luxembourg. The funders had no role in study design, data collection and analysis, decision to publish, or preparation of the manuscript.

## Author contributions

Investigation, S.A., M.M., E.K., and V.B. Writing – original draft, S.A. and M.M. Writing – review and editing, S.A., M.M., M.T., and E.L. Funding acquisition, M.T. and E.L. Supervision, M.T. and E.L. S.A. and M.M. contributed equally, and the order of the co-first authors’ names is determined alphabetically. Shared co-first authors reserve the right to list their names first on official personal documents.

## Declaration of interests

The authors declare no competing interests.
